# *ZNF821* as a potential biomarker associated with immune infiltration in pancreatic adenocarcinoma

**DOI:** 10.1590/1414-431X2026e14584

**Published:** 2026-07-10

**Authors:** Xiaocang Ren, Yanyan Ma, Jing Li, Yuee Liu, Zhihong Qiu

**Affiliations:** 1 Huabei Petroleum Administration Bureau General Hospital, Cangzhou, China

**Keywords:** ZNF821, Pancreatic adenocarcinoma, Methylation, Immune evasion, Immune infiltration

## Abstract

Pancreatic adenocarcinoma (PAAD) is a highly aggressive and lethal malignancy originating from the epithelial cells lining the pancreatic ducts. To date, no robust biomarkers have been established to reliably predict PAAD prognosis. To identify potential transcriptional biomarkers, we analyzed transcription factor expression in PAAD patients from The Cancer Genome Atlas (TCGA) and identified *ZNF821* as a novel prognostic marker. Higher *ZNF821* expression was significantly associated with improved patient survival and was positively correlated with increased immune infiltration and cytotoxic activity within the tumor microenvironment. Additionally, elevated *ZNF821* levels predicted a favorable response to immunotherapy. We further discovered that tumors downregulate *ZNF821* through DNA methylation, facilitating immune evasion during tumor progression. To investigate its functional mechanisms, we analyzed proteins regulated by or interacting with ZNF821. Moreover, we identified existing drugs that may target ZNF821, suggesting potential therapeutic applications for PAAD. Experimental validation using RT-PCR confirmed *ZNF821* downregulation in pancreatic cancer cell lines. Furthermore, tumor growth comparisons between *ZNF821*-overexpressing and ZNF821-null models demonstrated that ZNF821 enhances anti-tumor immunity, contributing to tumor suppression. In summary, our study identified *ZNF821* as a novel prognostic biomarker in PAAD, offering new insights into tumor immune evasion and potential therapeutic strategies.

## Introduction

Pancreatic adenocarcinoma (PAAD) is the seventh leading cause of cancer-related deaths worldwide, with a five-year survival rate of less than 10% (1,2). It is characterized by its late presentation, rapid progression, and poor prognosis (3). PAAD originates predominantly from the ductal epithelium of the pancreas and accounts for approximately 85% of all pancreatic cancer cases (4). Its dismal prognosis is primarily due to its late presentation and high propensity for metastasis, making early detection and effective treatment challenging (5-8).

The treatment of PAAD is multifaceted, typically involving surgery, radiation therapy, and chemotherapy. Surgical resection remains the only potential cure, yet only a small percentage of patients are eligible for surgery at diagnosis due to the advanced stage of the disease at presentation (9). The standard chemotherapy regimen includes agents like gemcitabine and nab-paclitaxel, or FOLFIRINOX (a combination of fluorouracil, leucovorin, irinotecan, and oxaliplatin), which offer modest improvements in survival but are associated with significant toxicity (10). For patients with locally advanced or metastatic disease, palliative care becomes the primary focus. This includes managing symptoms such as pain, jaundice, and weight loss, and improving quality of life. Palliative chemotherapy is used to extend life expectancy and alleviate symptoms in patients who cannot undergo surgery (11). In recent years, there has been an increasing focus on neoadjuvant therapies, that is, treatments given before surgery. These therapies, which often include combinations of chemotherapy and radiation therapy, aim to shrink tumors to a resectable size and eradicate microscopic disease, potentially increasing the success rate of surgical interventions and improving overall survival (12). Adjuvant therapies, administered post-surgery, seek to eliminate residual cancer cells and reduce the risk of recurrence. Recent advances have shown some promise in extending survival with the use of newer chemotherapeutic agents and combinations in the adjuvant setting (13). Checkpoint inhibitors that target PD-1/PD-L1 pathways have shown some efficacy in other types of cancer and are being tested in pancreatic cancer, though with limited success to date. This limited effectiveness might be due to the dense fibrotic stroma and immunosuppressive microenvironment characteristic of pancreatic tumors, which hinder immune cell infiltration and function (14).

Despite advancements in understanding and treating PAAD, significant challenges remain. The late diagnosis, complex tumor biology, and the dense stromal environment of pancreatic adenocarcinomas continue to hinder effective treatment outcomes. No robust biomarkers have been identified yet to predict the prognosis of PAAD, and the transcriptional mechanisms controlling PAAD immune microenvironment need to be further elucidated. Here, we analyzed the transcription factors (TFs) in PAAD patients and found a novel transcription factor ZNF821 to be an ideal biomarker for predicting prognosis in PAAD. We further compared the tumor growth between *ZNF821* overexpression and *ZNF821* null tumor model.

## Material and Methods

### Data acquisition

We retrieved gene expression data for PAAD from The Cancer Genome Atlas (TCGA) (https://portal.gdc.cancer.gov/repository) and corresponding survival information from UCSC (University of California Santa Cruz) Xena (https://xenabrowser.net/datapages/). The dataset includes 177 samples from 172 patients, with five patients having both tumor and adjacent normal tissue sequencing data. Among these patients, 94 are male and 78 are female. The majority of patients are classified as stage IIB (115/172, 66.9%), and most individuals in the cohort are white (151/172, 87.8%). Stage IIB in PAAD indicates locally advanced disease with involvement of regional lymph nodes but without distant metastasis, and is considered an intermediate to aggressive stage, associated with a poorer prognosis compared to early-stage tumors.

### DEG analysis between tumoral and healthy samples

We used Gene Expression Profiling Interactive Analysis (GEPIA) (http://gepia.cancer-pku.cn) (15) to perform differential expression (DE) analysis between tumor and normal samples in the PAAD dataset. The analysis was conducted using a threshold of false discovery rate (FDR)<0.05 and |log_2_(FoldChange)| >1. Additionally, we obtained a chromosome-wide distribution diagram of differentially expressed genes (DEGs) from the GEPIA website.

### Kaplan-Meier survival analysis

Kaplan-Meier survival analysis was conducted to examine the relationship between high and low *ZNF821* expression groups and patient survival outcomes. The analysis was performed using the Survival and Survminer R packages, and the results were visualized with a Kaplan-Meier curve using the ggsurvplot function in Survminer.

### Differentially expressed genes and functional enrichment assessment

DEGs between the two clusters were identified using the DESeq2 (16) package. A volcano plot was generated using the ggplot2 package, applying a false discovery rate (FDR) threshold of <0.05 and an absolute fold change >2. Gene Ontology (GO) and KEGG pathway enrichment analyses were performed on the top 200 DEGs (100 upregulated and 100 downregulated) using the clusterProfiler R package (17).

### Immune cell infiltration analysis

The proportions of various immune cell types in the tumor microenvironment were estimated using CIBERSORT via the IOBR (18) R package or TIMER2.0 (19) for a comprehensive assessment of immuno-oncology signatures. We also referenced the ICRAFT database (https://icraft.pku-genomics.org/) for information on immune checkpoint blockade (ICB) treatment responses.

### Protein-protein interaction network and TF-regulated proteins

To explore protein interactions associated with ZNF821, we used the STRING (20) database with an interaction score threshold of 0.15. Data on transcription factor-regulated proteins were retrieved from the TFDB database for further analysis.

### Cell culture and qRT-PCR

To verify the expression of *ZNF821* in pancreatic cancer, PANC-1 cells, 293T cells, and MCF-7 cells were obtained from ATCC. A mouse PAAD cancer cell line KPC cell line was purchased from KERAFAST (USA). PANC-1 and 293T cells were cultured in complete Dulbecco's Modified Eagle Medium (DMEM) (Gibco, USA), containing 10% fetal bovine serum (FBS) (Gibco) and 1% penicillin/streptomycin (Solarbio, China) at 37°C with 5% CO_2_. MCF7 and KPC cells were cultured in RPMI medium instead. After reaching a confluence of about 80% in a 6-well dish, total RNA was extracted using RNeasy Plus kits (Qiagen, Germany). cDNA was reversed transcribed using SuperScript III First-Strand Synthesis SuperMix (Invitrogen, USA) and then amplified on the QuantStudio 5 Real Time PCR System (Applied Biosystems, USA). Specific primers designed to amplify the gene of interest were combined with cDNA and SYBR Green qPCR SuperMix (Invitrogen, USA). qPCR was done with the following method: incubation at 50°C for 2 min, incubation at 95°C for 2 min, 40 cycles at 95°C for 10 s, incubation at 60°C for 30 s, and a final step for melting curve generation. Values were normalized to β-actin expression. Primers used in this study were: *ZNF821*: (F) CCCGGGTCCATTGTCCGGCG, (R) GGTGCAGTTCCCACGGAGC; *β-Actin*: (F) TCCCTGGAGAAGAGCTACG, (R) GTAGTTTCGTGGATGCCACA.

### Mouse tumor model construction and overexpression of ZNF821 in cancer cells

Lentivirus was packaged for the overexpression of *ZNF821*. pLVX-*ZNF821* (5 µg), 3 µg pSPAX2, and 2 µg pMD2G were transfected in 293T cells in a 10-cm dish with a confluence of 80%. Viruses were harvested after 48 h and stored at -80°C for further use. KPC cells were seeded onto 6-well plates to reach a confluence of about 30%. One mL virus was added and allowed infection for 12 h. After 48 h, cells with expression of *ZNF821* were sorted by the puromycin selection of 5 µg/mL. Ten million KPC cells were injected subcutaneously into both wild type C57BL6N mice and TCR-/- mice. Tumor growth was monitored every five days. A total of three mice were used in each group. For each tumor, measurements were taken twice per time point. Tumor volume was calculated using the widely used formula: 1/2 × length × width^2^.

The *Tcra* knockout strain and wild type C57BL/6J mice were obtained from the Jackson Laboratory (USA). Animal care, feeding, housing, and enrichment were accomplished in the Shang Hai MODEL ORGANISMS (China). All the surgeries on the mice were carried out under isoflurane anesthesia and carbon dioxide euthanasia was used to sacrifice the mice. Four 8-week-old mice were included in each group. All animal-related experiments were reviewed and approved by the Shanghai Model Organism Ethics Committee.

### Immunohistochemistry (IHC)

Tumor tissues from both the vector control and *ZNF821* overexpression groups were collected, fixed in 10% neutral-buffered formalin, and embedded in paraffin. Tissue sections (5 µm) were deparaffinized, rehydrated through a graded ethanol series, and subjected to antigen retrieval using Tris-EDTA (pH 9.0). After blocking with 5% BSA at room temperature for 30 min, sections were incubated overnight at 4°C with phycoerythrin (PE)-conjugated anti-CD8 antibody (Sigma, 1:1000, USA) to visualize CD8+ T cells. Nuclei were counterstained with DAPI (Sigma, 1:1000). Stained sections were imaged using a fluorescence confocal microscope (Nikon Eclipse Ti2-E, Japan).

### Single-cell RNA-seq analysis

We analyzed the single-cell RNA-seq dataset GSE205013 (21), which contains tumor microenvironment profiles of PAAD. Raw count data were processed using Seurat (v4.3.0) in R (22). Cells were filtered based on mitochondrial gene expression and total UMI count. Data were normalized, scaled, and integrated using Harmony for batch correction. Dimensionality reduction was performed using UMAP, and clusters were identified by unsupervised Louvain algorithm. Cell type annotation was based on canonical markers and validated by differential gene expression. *ZNF821* expression was visualized using feature and dot plots.

## Results

### Identification of transcription factors in PAAD

**Figure 1:**
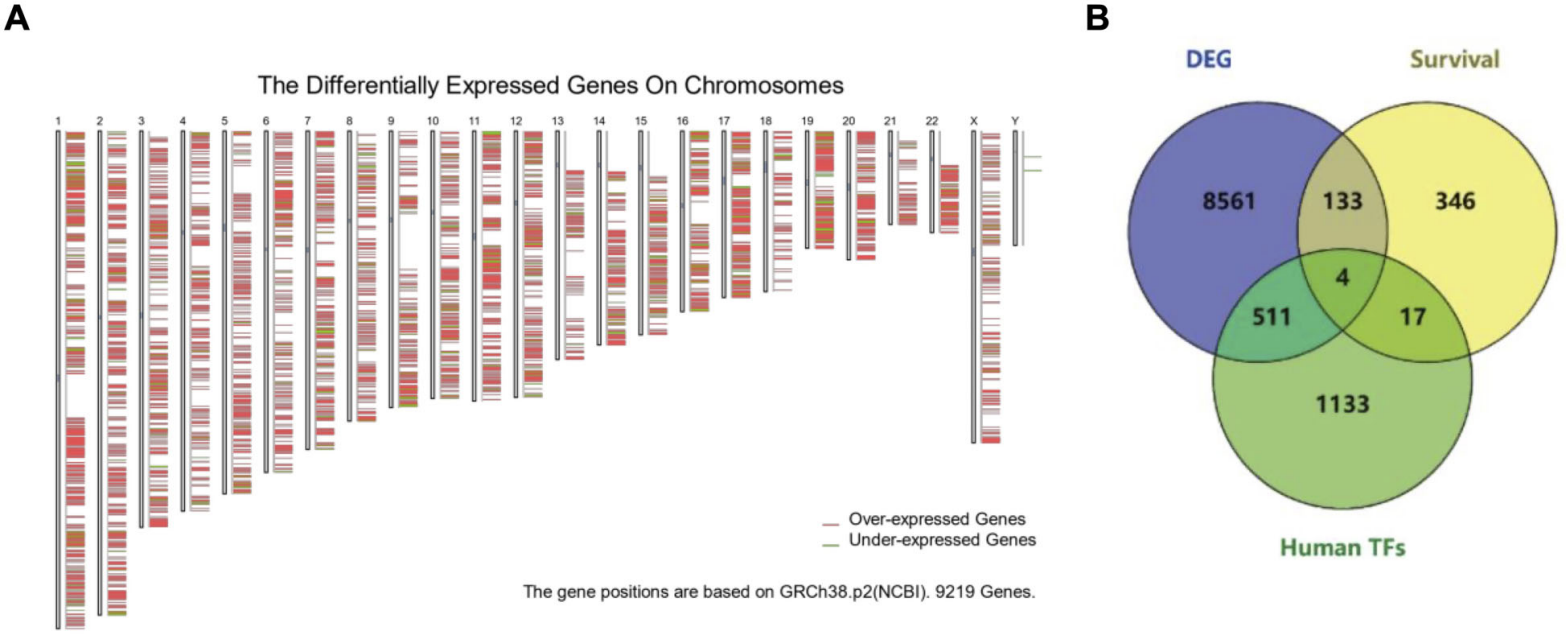
Identification of transcription factors in pancreatic adenocarcinoma (PAAD). **A**, Distribution of differentially expressed genes (DEGs) in chromosomes. **B**, Human transcription factors (TFs) intersected with DEGs, and genes correlated with survival (by Cox regression).

Firstly, we compared the DEGs between tumor and normal tissue and identified 9219 DEGs (FDR<0.05, |log2(FoldChange)|>1). They were widely distributed across all chromosomes (Figure 1A). Notably, chromosome 19 showed the highest enrichment of DEGs per million bases. To identify key TFs within these DEGs, we cross-referenced the DEGs with known human TFs and genes associated with survival. We performed survival analysis based on overall survival (OS) data rather than focusing solely on mortality. Unlike binary mortality outcomes, survival analysis provides a time-to-event perspective, enabling a more detailed evaluation of gene expression patterns associated with longer or shorter survival durations. This is particularly important in PAAD, where disease progression and patient prognosis can vary widely. We ultimately identified four critical TFs (Figure 1B): ARNTL2, NFE2L3, ZFP91, and ZN821. While ARNTL2, NFE2L3, and ZFP91 are well-documented in their association with PAAD prognosis (23-25), ZN821 is a newly discovered TF linked to the disease's prognosis. Consequently, we focused on ZN821 in further study to comprehensively elucidate its role and mechanism in regulating PAAD progression.

### PAAD subtypes based on *ZNF821* expression

**Figure 2:**
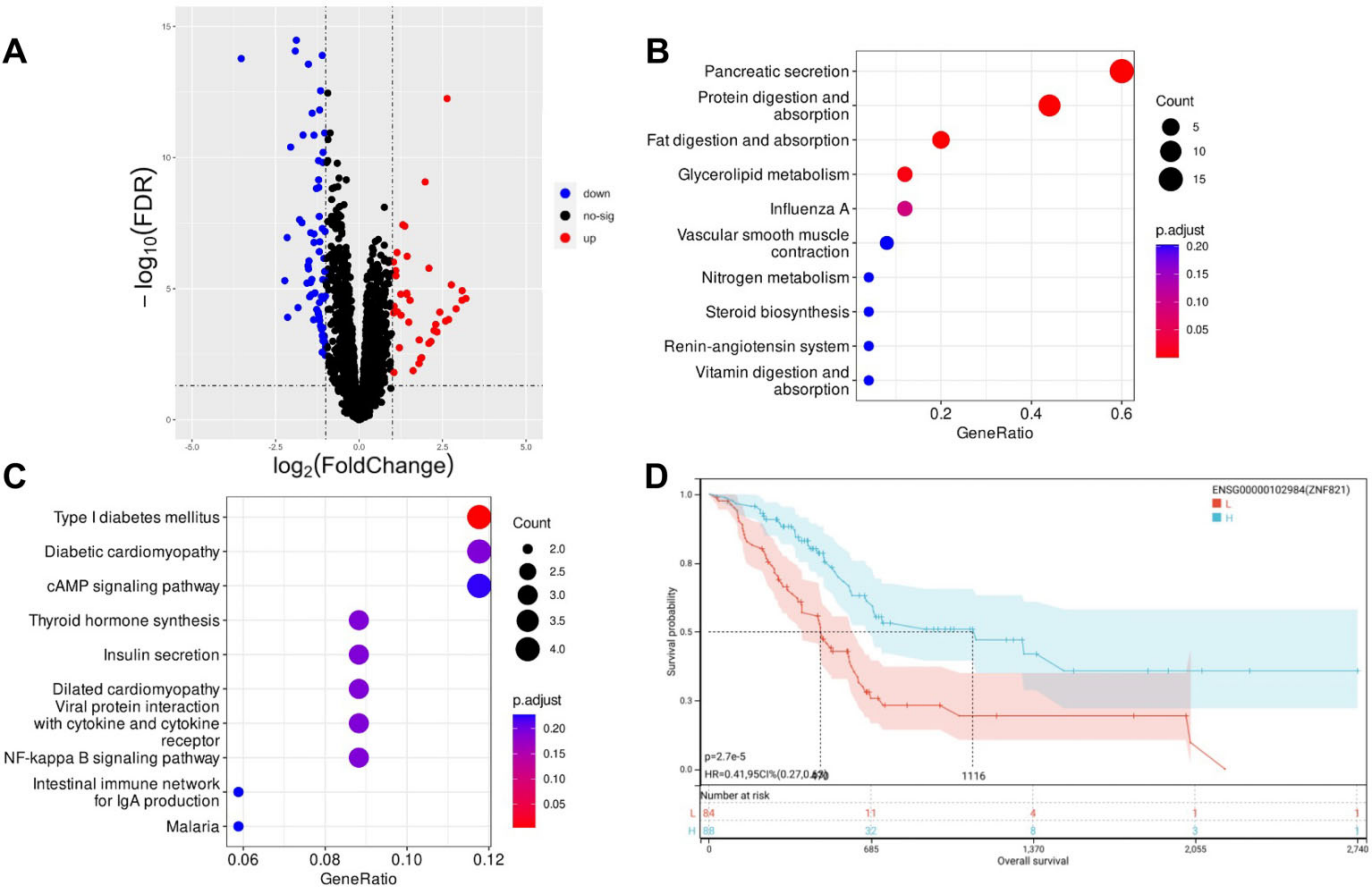
Pancreatic adenocarcinoma (PAAD) subtypes based on ZNF821 expression. **A**, Volcano plot showing differentially expressed genes (DEGs) between patients of different ZNF821 expression. **B**, KEGG enrichment of upregulated DEGs in high ZNF821 patient group. **C**, KEGG enrichment of downregulated DEGs in low ZNF821 patient group. **D**, Survival curve of the two subtypes. H: high; L: low. P<0.0001, log-rank test.

To further investigate ZNF821's role in PAAD, we classified patients into two subtypes based on median ZNF821 expression levels. Patients with expression above the median were assigned to the high-expression subtype. We analyzed the DEGs between these subtypes, identifying 39 upregulated genes and 83 downregulated genes in ZNF821-high subtype (Figure 2A). Subsequent KEGG pathway enrichment analysis revealed that most upregulated genes in ZNF821 high patient group were involved in pancreatic secretion, protein digestion, absorption pathways, and influenza A infection. In contrast, the downregulated genes were predominantly related to pathways of type I diabetes mellitus and diabetic cardiomyopathy (Figure 2B and C). These findings suggest that patients with higher *ZNF821* expression may have better pancreatic secretion and protein digestion functions, as well as improved immune response. Moreover, patients with elevated ZNF821 levels demonstrated a significantly better prognosis (Figure 2D). Thus, varying ZNF821 expression levels correlate with distinct prognosis and pancreatic functions, underscoring ZNF821's crucial role in PAAD progression.

### ZNF821 had a positive correlation with immune infiltration

**Figure 3:**
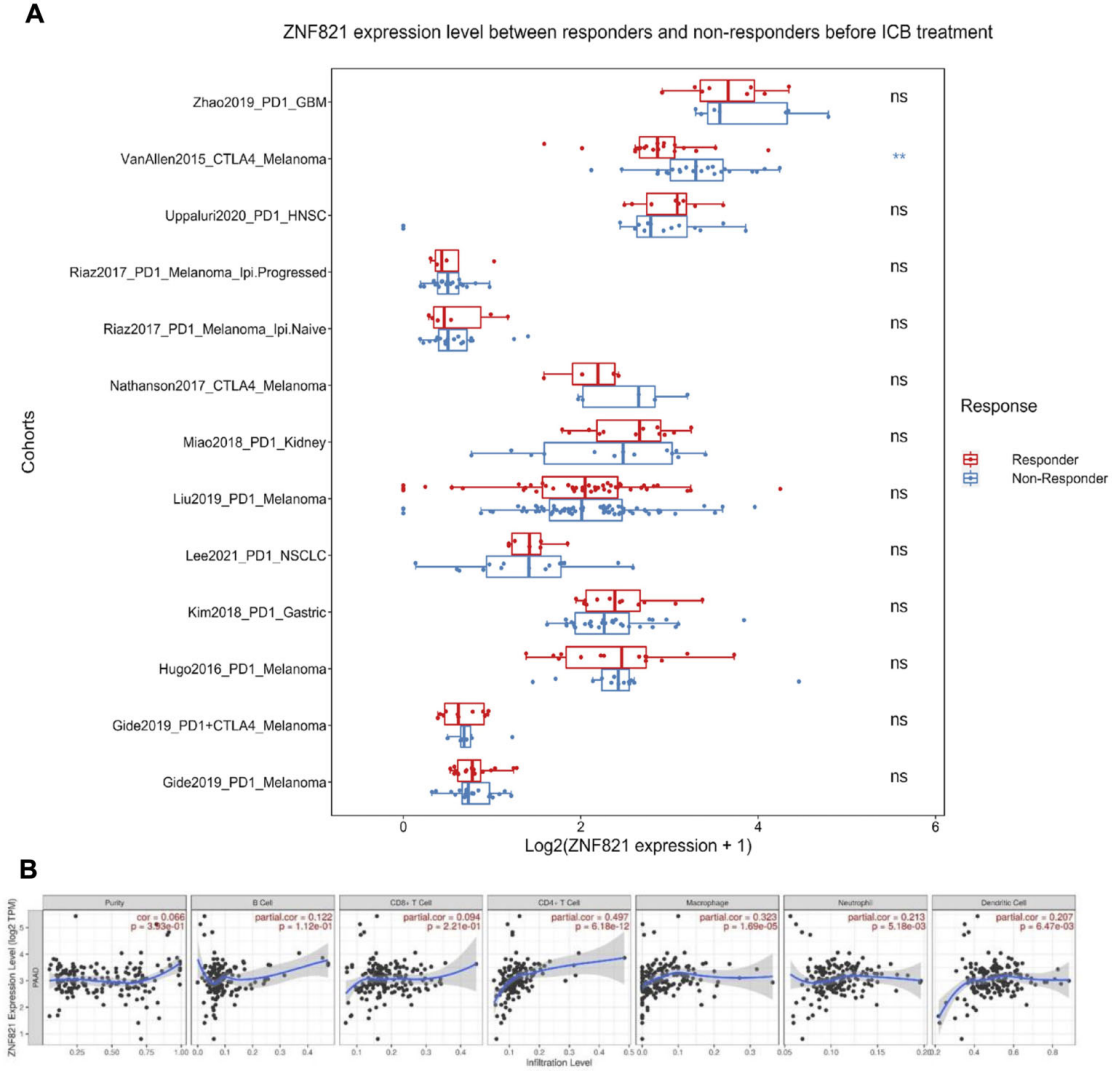
ZNF821 had a positive correlation with immune infiltration, **A**, ZNF821 was correlated with immune checkpoint blockade (ICB) response in some cancers. Data are reported as median and interquartile range. P<0.01, *t*-test; ns: non-significant. **B**, Infiltration of the main immune cell types correlated with ZNF821 expression in pancreatic adenocarcinoma.

Some of the upregulated DEGs were enriched in immune-related pathways, such as influenza infection (Figure 2B), suggesting a potential role for ZNF821 in modulating immune responses. Given this observation of enhanced immune infiltration, we investigated whether ZNF821 influences the response to ICB therapy. Using the ICRAFT platform, which compiles publicly available datasets, we found that *ZNF821* expression is associated with improved response to CTLA-4 blockade in melanoma (Figure 3A), indicating a possible link between ZNF821 and ICB responsiveness across cancers. Further analysis demonstrated that *ZNF821* expression positively correlates with the infiltration of key immune cell types in the tumor microenvironment - including B cells, dendritic cells (DCs), T cells, macrophages, and neutrophils - all of which showed significantly increased levels (Figure 3B). These immune cells play central roles in tumor surveillance and immune regulation, highlighting a potential role for ZNF821 in promoting the infiltration of beneficial immune populations into the tumor microenvironment.

### ZNF821 was related to cytotoxicity and ICB response in PAAD

**Figure 4:**
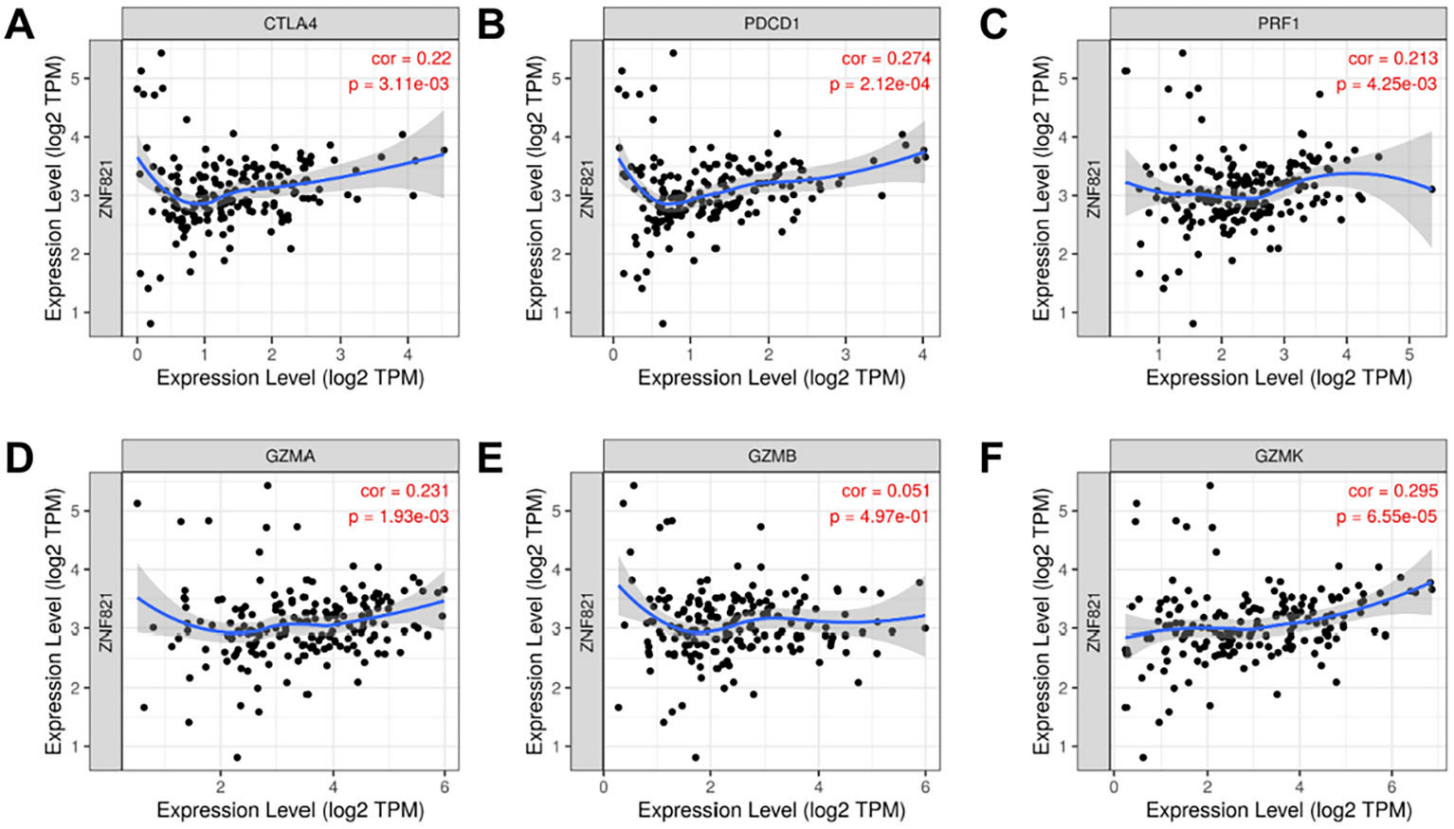
ZNF821 was correlated to cytotoxicity and immune checkpoint blockade (ICB) response in pancreatic adenocarcinoma. **A**-**F**, CTLA4, PDCD1, PRF1, GZMA, GZMB, GZMK were positively correlated with ZNF821expression. P<0.05, Pearson's correlation.

To predict the response of ICB therapy in PAAD, we measured the expression level of PD1 and CTLA4 in PAAD. Both CTLA4 and PD1 were significantly correlated with the expression of *ZNF821*, showing ZNF821's positive correlation with response to ICB therapy (Figure 4A and B). We further analyzed the genes related to cytotoxicity of T cells and NK cells in patients. Consistently, higher *PRF1*, *GZMA*, *GZMB*, and *GZMK* were all significantly correlated with higher ZNF821 expression (Figure 4C-F), which demonstrates ZNF821's potential role in regulating cytotoxic cell function.

### Methylation of *ZNF821* promoter in PAAD during tumor progression

**Figure 5:**
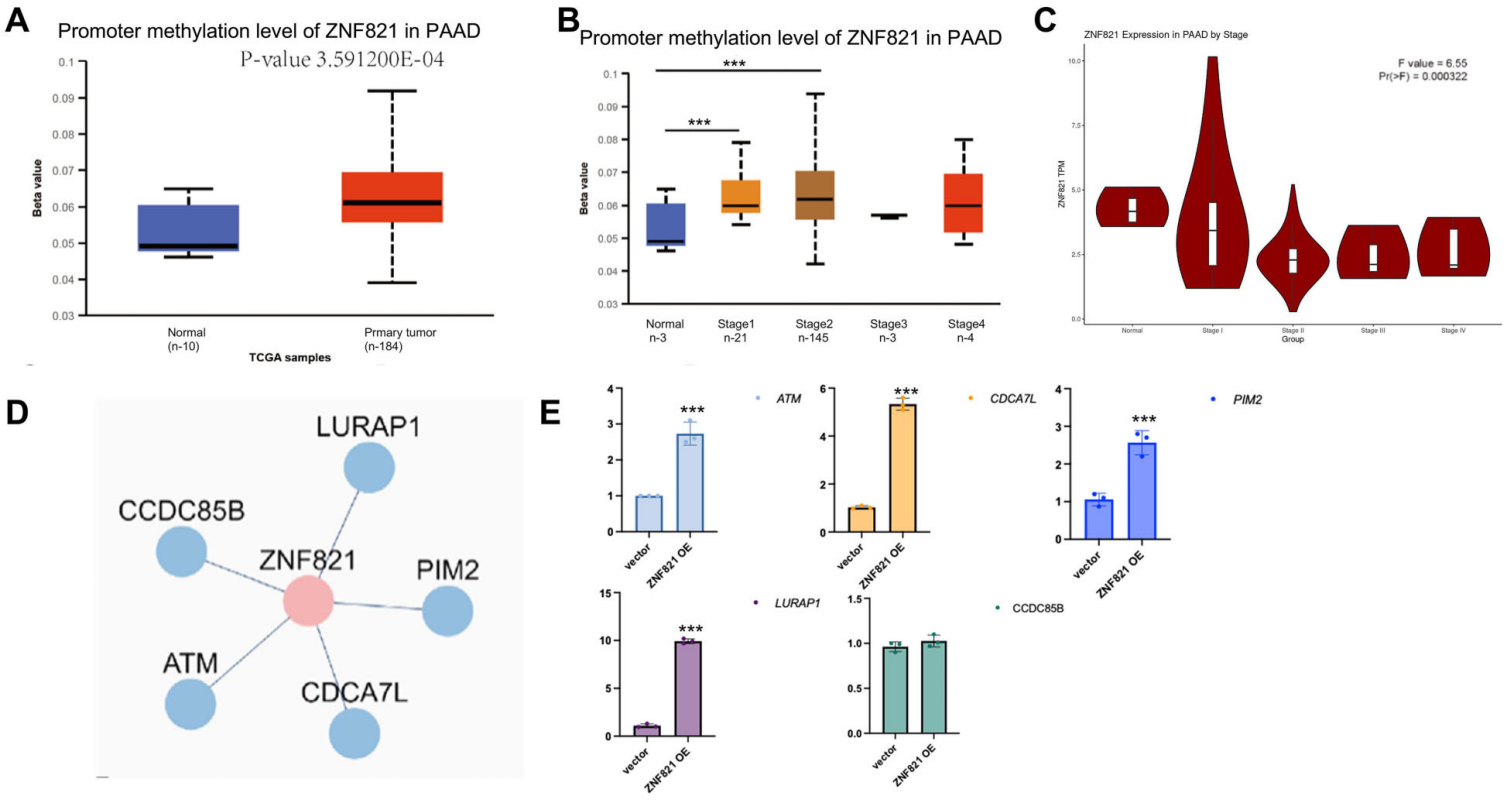
Regulation mechanism of ZNF821 in pancreatic adenocarcinoma (PAAD). **A**, ZNF821 promoter methylation levels between tumor and normal tissue in PAAD. **B**, ZNF821 promoter methylation levels at different stages in PAAD. Data are reported as median and interquartile range. ***P<0.01, *t*-test. **C**, ZNF821 expression level at different stages of PAAD. **D**, Down-stream genes regulated by ZNF821. **E**, *ATM*, *CDCA7L*, *PIM2*, and *LURAP1* showed elevated RNA level after over-expression of *ZNF821*, while *CCDC85B* showed no significant mRNA level changes. Data are reported as mean and SD. ***P<0.05, *t*-test.

The positive correlation between ZNF821 and immune infiltration is unfavorable for tumor development. Therefore, PAAD downregulated *ZNF821*. To understand how tumors effectuate this change, we investigated ZNF821's promoter methylation across various tumor stages. We discovered a significant increase in *ZNF821* promoter methylation coinciding with a decrease in *ZNF821* expression as tumors progressed (Figures 5A-C). These observations potentially explain the mechanism of PAAD's immune evasion during disease progression. We then analyzed the down-stream regulated proteins and interacting proteins of ZNF821 by TFDB and STRING databases. Genes regulated by ZNF821 include genes controlling cell division and pro-inflammatory genes like *ATM* and *LURAP1* (Figure 5D). We further did a qPCR of KPC cells to verify the regulation of these downstream genes. *ATM*, *CDCA7L*, *PIM2*, and *LURAP1* showed elevated RNA levels after over-expression of ZNF821, while *CCDC85B* showed no significant mRNA level changes (Figure 5E). These findings revealed ZNF821's potential mechanisms in regulating PAAD immune microenvironment.

### Structure and potential targeted drugs of ZNF821

To demonstrate ZNF821's potential in drug development, we investigated drugs that could activate ZNF821 to enhance clinical outcomes in PAAD using the DSigDB database (26). Three drugs were identified as potential activators of ZNF821 (Table 1). Among these drugs, MG132 was proven to be able to stabilize Zinc finger proteins, thus is predicted to strengthen the function of ZNF821 (27). Also, ZNF821 was upregulated following the cephaeline small molecule perturbation from the CMAP Signatures of Differentially Expressed Genes for Small Molecules dataset (28). These findings could be highly valuable for clinical trials.

**Table 1:** Potential targeted drugs of ZNF821, three of which have activation function (in bold type).

Gene	Source	Chemical Name
*ZNF821*	D3(DOWN)	0175029-0000
*ZNF821*	D3(UP)	**cephaeline**
*ZNF821*	D3(UP)	**MG132**
*ZNF821*	D3(UP)	**puromycin**
*ZNF821*	D4 CTD	ZINC
*ZNF821*	D4 CTD	4-Hydroxytamoxifen
*ZNF821*	D4 CTD	estradiol
*ZNF821*	D4 CTD	Premarin

### Verification of *ZNF821*'s expression and anti-tumor function in PAAD

**Figure 6:**
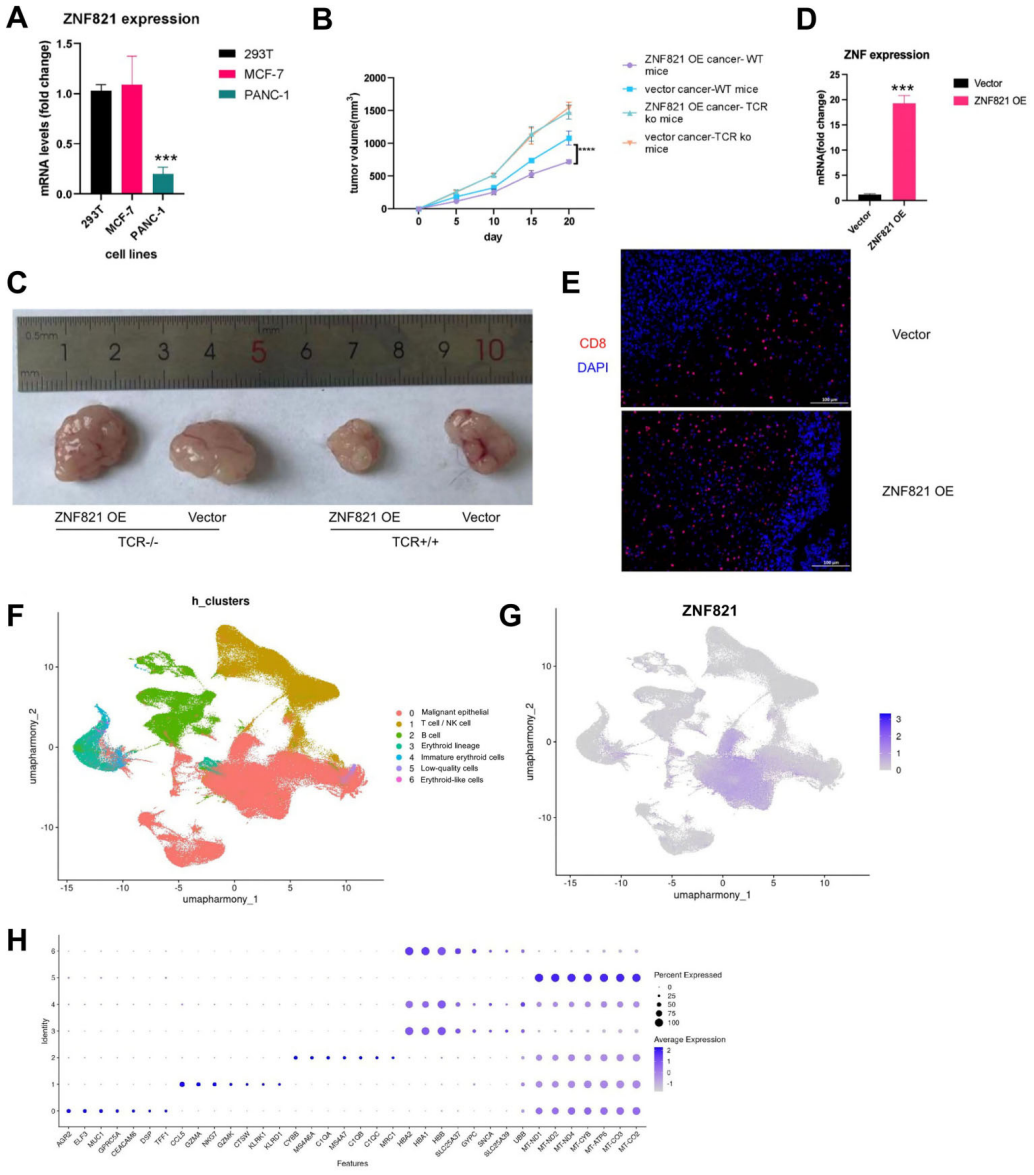
Verification of ZNF821's expression and anti-tumor function in pancreatic adenocarcinoma (PAAD). **A**, ZNF821 mRNA level was significantly lower in PANC-1 compared to other cancer cell lines or non-cancer cell lines. Data were analyzed using the comparative Ct method, normalized to β-actin expression. ***P<0.001. **B**, Tumor volumes of each group. ****P<0.0001, Student's *t*-test. **C**, Representative photographic image of tumors of the four groups. **D**, qPCR of verification of ZNF821 overexpression. ZNF821 had a 20 times higher mRNA level compared to the vector group. Data are reported as mean and SD. ***P<0.05, *t*-test. **E**, Representative immunohistochemistry image showing increased CD8 T cell infiltration in ZNF821 OE tumors. CD8 was labeled by PE (red), and the nucleus was labeled by DAPI (blue). **F**, UMAP visualization of cell clusters from GSE205013 dataset, with major cell types annotated. **G**, Feature plot showing ZNF821 expression across all clusters. ZNF821 is predominantly expressed in malignant epithelial cells, with minimal or no expression in T cells or other immune cells. **H**, Dot plot showing the average expression and proportion of expressing cells for selected marker genes across clusters, supporting cluster annotation. WT: wild type; ko: knock-out, embedding generated after applying Harmony batch correction/integration.

To validate *ZNF821*'s expression in pancreatic cancer, we conducted a qRT-PCR of pancreatic cancer cell line PANC-1. Breast cancer cell line MCF-7 and non-cancer cell line 293T were used as controls. We found that PANC-1 had a significantly lower expression of ZNF821 compared to control cell lines (Figure 6A). We next compared the tumor growth between *ZNF821* overexpression cancer cell and *ZNF821* null cell. We found that ZNF821 expression can lead to slower tumor growth and this effect depends on T cells (Figure 6B and C). To be certain of our findings, we did a qPCR and found that the relative expression of ZNF821 increased about 20 times compared with vector tumor cells (Figure 6D). We further did an immunohistochemistry (IHC) analysis to verify the upregulated level of CD8 T cell infiltration by ZNF821 overexpression (Figure 6E). The results showed that a section of ZNF821 tumor sample had significantly more infiltrated CD8 T cells compared with vector control. To determine the cellular source of ZNF821 within the tumor microenvironment, we analyzed single-cell RNA-seq data from the GSE205013 dataset. Cell clustering and annotation revealed several major populations, including malignant epithelial cells, T/NK cells, B cells, and erythroid lineage cells (Figure 6F). ZNF821 expression was almost exclusively restricted to the malignant epithelial (tumor) cells (Figure 6G) and was not detected in T cells or other immune cells. This suggests that the protective effect of ZNF821 on anti-tumor immunity is likely indirect, rather than mediated through direct expression in CD8^+^ T cells. Cluster identities were confirmed using marker gene expression profiles (Figure 6H). These results showed that ZNF821 can promote anti-tumor T cell immunity in the PAAD model. Our findings suggest that downregulation of ZNF821 might be a potential immune evasion mechanism in pancreatic cancer.

## Discussion

In this study, we first investigated the TFs corrected to survival in PAAD and selected a novel gene - *ZNF821*. Unlike many previous studies that primarily focus on upregulated genes, our strategy involved analyzing the full set of DEGs regardless of direction and intersecting them with known transcription factors and prognostic markers. This comprehensive intersection allowed us to identify ZNF821, a downregulated transcription factor, which may have been overlooked in studies focusing only on genes with increased expression. We then did a pan-cancer analysis and found that ZNF821 may play a role in a wide range of cancers. We further focused on PAAD to explore the role of ZNF821. Subtypes based on the expression of ZNF821 showed that patients with higher ZNF821 may have better pancreatic secretion function and immune response. Also, patients with higher ZNF821 had a much better prognosis and immune infiltration. Mechanically, ZNF821 may function by regulating down-stream genes including *ATM*, *CDCA7L*, *PIM2*, and *LURAP1*.

*ZNF821* encodes a protein with two C2H2 zinc finger motifs and a score-and-three-amino acid peptide repeat (STPR) domain. The STPR domain of the encoded protein binds to double-stranded DNA and may also contain a nuclear localization signal, suggesting that this protein interacts with chromosomal DNA. This gene is poorly studied and its function remains largely unknown. Some researchers have reported that, in the context of synovial sarcoma, *ZNF821* mutations were identified in metastatic lesions, suggesting a role in disease progression (29).

Among the downstream genes regulated by ZNF821, *ATM* is a tumor suppressor gene that controls the rate of cell growth and division, helping to protect against breast, prostate, and pancreatic cancers (30). *CDCA7L* is also involved in cell division (31). *PIM2* (proviral integration site for Moloney murine leukemia virus) kinase is part of the PIM family of serine/threonine kinases - including *PIM1*, *PIM2*, and *PIM3* - which are known for their roles in regulating cell survival, proliferation, and differentiation (32). *LURAP1* is involved in dendritic cell-mediated immune responses and plays a role in inflammatory signaling pathways (33). Therefore, most of the downstream genes regulated by ZNF821 are involved in cell cycle control and immune responses, suggesting that ZNF821 fine-tunes the tumor microenvironment by transcriptionally regulating a series of genes related to proliferation and immunity.

While our study identified *ZNF821* as a potential prognostic biomarker and therapeutic target in PAAD, several limitations should be acknowledged. First, our findings are primarily based on bioinformatic analyses using publicly available datasets, which, while comprehensive, require further experimental validation in larger and independent patient cohorts. Second, although we demonstrated the downregulation of *ZNF821* in pancreatic cancer cell lines and its potential functional role in tumor suppression, *in vivo* studies using patient-derived xenografts or genetically engineered mouse models are necessary to confirm its impact on tumor growth and immune response. Additionally, while our analysis suggests that *ZNF821* is regulated by promoter methylation, further mechanistic studies are needed to fully elucidate the epigenetic and transcriptional pathways controlling its expression. Finally, our identification of potential drugs targeting ZNF821 is based on predictive modeling and requires experimental validation to assess their efficacy in PAAD treatment. Despite these limitations, our study provides a strong foundation for future investigations into the clinical relevance of ZNF821 in PAAD and beyond.

In summary, our study highlights the significance of *ZNF821* in predicting prognosis and immune infiltration in PAAD. We also propose a novel immune evasion mechanism in PAAD involving hypermethylation of the *ZNF821* promoter. Our findings offer new insights into PAAD pathology and potential therapeutic strategies.

## Data Availability

All data generated or analyzed during this study are included in this published article.
